# ESCO2 inhibition induces cell cycle arrest and apoptosis in breast cancer via the P53-CDK1 axis and the BAX/Bcl2/caspase signaling cascade

**DOI:** 10.3389/fonc.2025.1585945

**Published:** 2025-07-10

**Authors:** Pingchuan Li, Lineng Wei, Meng Li, Xiaoqiang Liu, Huawei Yang

**Affiliations:** Department of Breast Surgery, Key Laboratory of Breast Cancer Diagnosis and Treatment Research of Guangxi Department of Education, Guangxi Medical University Cancer Hospital, Nanning, China

**Keywords:** ESCO2, breast cancer, cell cycle, apoptosis, p53

## Abstract

**Background:**

Breast cancer is a major threat to women’s health, and dysregulation of the cell cycle is a critical driver of its progression. ESCO2, a potential key regulator of the cell cycle, is implicated in cancer development; however, its specific role and mechanisms in breast cancer remain poorly understood.

**Methods:**

We analyzed differentially expressed genes between breast cancer and normal breast samples from GEO datasets to identify potential key regulators of the cell cycle pathway. ESCO2 expression was further investigated in breast cancer cell lines. Functional assays, including overexpression and knockdown of ESCO2 in MDA-MB-231 and MDA-MB-468 cells, were performed to assess its effects on the cell cycle and apoptosis. Molecular mechanisms were explored using Western blot, and rescue experiments were conducted to validate key regulatory pathways.

**Results:**

Analysis of the GSE38959 and GSE70947 datasets identified 541 common differentially expressed genes, with 26 genes enriched in the cell cycle pathway. ESCO2 interacted with multiple cell cycle-related genes and was significantly overexpressed in breast cancer. Overexpression of ESCO2 promoted DNA replication, while its knockdown induced G2/M phase arrest via the ESCO2-P53-CDK1 regulatory axis, and triggered apoptosis through the BAX/Bcl2/caspase9/caspase7 signaling cascade. The effects of ESCO2 knockdown on the cell cycle and apoptosis were rescued by siP53.

**Conclusion:**

Our findings reveal that ESCO2 is upregulated in breast cancer and may contribute to cell cycle regulation and apoptosis through the p53-CDK1 and BAX/Bcl-2-caspase pathways. These results highlight ESCO2 as a potential therapeutic target and provide new mechanistic insights into breast cancer progression.

## 1 Introduction

Globally, breast carcinoma (BRCA) stands as one of the most widespread and aggressive cancers impacting the female population, consistently ranking as the primary contributor to disease burden among all cancers affecting women (1). Due to the high heterogeneity of breast cancer patients, traditional treatment methods often struggle to achieve true personalized and precision medicine. Thus, the quest for potential molecular markers and therapeutic targets in BRCA remains a critical area of ongoing research (2).

The cell cycle is a fundamental process of cell growth, division, and proliferation. Proper regulation of the cell cycle is crucial for maintaining cellular function and life activities (3). However, the regulation of the cell cycle is often disrupted in cancer cells, leading to abnormal cell proliferation and tumor formation (4). In breast cancer, dysregulation of the cell cycle is closely associated with tumor initiation and progression (5). Therefore, molecules involved in cell cycle regulation have become potential therapeutic targets in breast cancer treatment. Research on key cell cycle regulatory proteins, such as CDK4/6 inhibitors, has provided new insights into the development of novel therapeutic strategies for breast cancer (6).

The establishment of sister chromatid cohesion by N-acetyltransferase 2 (ESCO2), a homolog of sister chromatid cohesion proteins, is critical during the S phase of the cell cycle (7). In recent years, research on the role of ESCO2 in cancer has gradually increased, primarily focusing on its impact on DNA repair and chromosome stability (8, 9). In addition, ESCO2 is also involved in regulating the cell cycle and apoptosis of colorectal cancer and gastric cancer (10, 11).

However, the specific biological functions and mechanisms of ESCO2 in breast cancer have not been thoroughly studied. We conducted a comprehensive bioinformatics analysis of breast cancer samples from the GEO database, which gradually identified ESCO2 as a potential key molecule in the cell cycle. Additionally, the expression differences of ESCO2 were validated through multiple breast cancer cell lines. Through cell functional experiments, we further determined the regulatory potential and mechanisms of ESCO2 in the cell cycle and apoptosis of breast cancer cells. Our results reveal that ESCO2 could be a valuable therapeutic candidate in breast cancer patients, providing new insights for clinical treatment strategies in breast cancer.

## 2 Materials and methods

### 2.1 Bioinformatics analysis

The sequencing data from the GSE38959 and GSE70947 datasets were obtained from the GEO database (https://www.ncbi.nlm.nih.gov/geo/). Differential gene expression analysis, KEGG pathway enrichment analysis, and visualization were performed using R 4.2.2. Protein-protein interaction networks were constructed using the STRING database (https://string-db.org/). Single-gene functional enrichment analysis was conducted using GSEA 4.3.1 software.

### 2.2 Cell culture

The human breast cancer cell lines MCF-7 (ER+/HER2-), BT-474 (HER2+), SK-BR-3 (HER2+), MDA-MB-231 (triple-negative), and MDA-MB-468 (triple-negative) were cultured in Dulbecco’s Modified Eagle Medium (DMEM, Gbico, USA) supplemented with 10% fetal bovine serum (FBS, WISENT, Canada) and 1% penicillin-streptomycin. The normal mammary epithelial cell line MCF-10A was cultured in specific MCF-10A growth medium (Pricella, China). All cells were maintained in a humidified incubator at 37°C with 5% CO_2_. The culture medium was replaced every 2–3 days, and cells were passaged at 80-90% confluence using 0.25% trypsin-EDTA. All cell lines were authenticated by short tandem repeat (STR) profiling at American Type Culture Collection (ATCC) and confirmed to be free of mycoplasma contamination. All experiments were performed with cells at passages 5-20.

### 2.3 RT-qPCR

Total RNA was extracted using RNAiso Plus (TaKaRa, Japan), and cDNA was synthesized with PrimeScript RT kit (TaKaRa, Japan). Real-time qPCR was performed using SYBR Green mix (Roche, Switzerland) on a MyiQ real-time PCR detection system (Bio-Rad, USA).Relative mRNA levels were calculated using the 2^-ΔΔCt method. The primer sequences used were as follows:

ESCO2 F’: 5’-TGGGATAAGTAGAATCTGGGTT-3’;ESCO2 R’: 5’-ATACGAGGAAATTAGGGGTGT-3’;GAPDH F’:5’-CTCTGCTCCTCCTGTTCGAC-3’;GAPDH R’:5’-TTAAAAGCAGCCCTGGTGAC-3’.

### 2.4 Western blot

Cells were lysed in RIPA buffer (Solarbio, China). Proteins (50 μg per lane) were separated by SDS-PAGE, transferred to PVDF membranes, and incubated with primary antibodies, followed by HRP-conjugated secondary antibodies. Signals were detected via enhanced chemiluminescence (ECL). The antibodies used in this study are shown in **Table 1**. Each band underwent 3 repeated experiments. Normalize the grayscale values and perform statistical analysis.

**Table 1:** Antibody list.

Name	Company	Dilution ratio	Source	Cat. No.
ESCO2	Novus Biologicals	1:5000	Rabbit	NB100-87021
CDK1	Zenbio	1:1000	Rabbit	R23884
CyclinB1	Zenbio	1:1000	Rabbit	340296
Bcl2	Abmart	1:2000	Rabbit	T40056
caspase7	Proteintech	1:1000	Rabbit	27155-1-AP
caspase9	Abcam	1:1000	Rabbit	ab32539
P53	Santa Cruz	1:500	Mouse	sc-393031
p-p53	Zenbio	1:1000	Rabbit	310029
p21	Abmart	1:2000	Rabbit	T55543
BAX	ABclonal	1:1000	Rabbit	A12009
GAPDH	Servicebio	1:1000	Rabbit	GB15004-100
beta-Actin	Servicebio	1:1000	Rabbit	GB15003-100
Vinculin	Abclonal	1:5000	Rabbit	A2752
Goat anti-mouse IgG-HRP	Beyotime	1:5000	Goat	A0350
Goat anti-rabbit IgG-HRP	Beyotime	1:5000	Goat	A0352

### 2.5 Cell transfection

The shESCO2 Lentiviruses was obtained from the GeneChem company. Lentiviruses overexpressing ESCO2 was purchased from GenePharma. Add virus solution when the cell fusion degree is 30-50%, replace the complete culture medium after 24 hours, and add puromycin for screening after 48 hours. The sequence used for transfection was:

Vector: 5’-CCGGGCAGCTTTTTTG-3’; OE: NM_001017420.3 CDS area(NCBI Reference Sequen-ce); shNC:5’-TTCTCCGAACGTGTCACGT-3’; shESCO2: 5’-GCAAGTCTTGTGGTATGATAT-3’.

The siRNA was obtained from the Haixing Biosciences at a final concentration of 50nM. Perform cell transfection according to lipo6000 instructions (Beyotime). The sequence wsa as follows:

siNC: 5’-GCTTCGCGCCGTAGTCTTA-3’; siP53: 5’-GACUCCAGUGGUAAUCUAC-3’.

### 2.6 Cell cycle detection

Cell cycle distribution was analyzed using the KeyGen BioTECH Cell Cycle Detection Kit according to the manufacturer’s instructions. Briefly, breast cancer cells stably overexpressing or knockdown for ESCO2 were seeded in 6-well plates at a density of 2 × 10^5^ cells per well and allowed to adhere overnight. When the cell confluence reaches around 80%, cells were harvested by trypsinization, washed twice with cold phosphate-buffered saline (PBS), and fixed in 70% ice-cold ethanol at 4°C overnight. Fixed cells were then centrifuged at 1,000 × g for 5 minutes, and the ethanol was carefully removed. Cells were washed once with PBS and resuspended in 500 µL of staining solution containing propidium iodide (PI) and RNase A, prepared according to the kit protocol. The cell suspension was incubated in the dark at 37°C for 30 minutes. Cell cycle distribution was analyzed using CytoFLEX flow cytometer, and data were processed using ModFit LT 5. The percentages of cells in the G0/G1, S, and G2/M phases were determined based on DNA content.

### 2.7 Apoptosis analysis

Apoptosis was assessed using the Annexin V-APC/7-AAD Apoptosis Detection Kit (MULTI SCIENCE) according to the manufacturer’s instructions. Briefly, breast cancer cells stably overexpressing or knockdown for ESCO2 were seeded in 6-well plates at a density of 2 × 10^5^ cells per well and allowed to adhere overnight. When the cell confluence reaches around 80%, cells were harvested by gentle trypsinization, washed twice with cold PBS, and resuspended in 1× binding buffer at a concentration of 1 × 10^6^ cells/mL. A total of 100 µL of cell suspension was incubated with 5 µL of Annexin V-APC and 10 µL of 7-AAD in the dark at room temperature for 15 minutes. After incubation, 400 µL of 1× binding buffer was added to each sample, and the cells were analyzed immediately using CytoFLEX flow cytometer. Data were processed using FlowJo V10.

### 2.8 CCK-8 assay

The cell density was standardized to 2 × 10^4^ cells/mL, and each well of a 96-well plate was inoculated with 100 μL of the suspension. At designated time points (0, 24, 48, 72, and 96 h), the spent medium was exchanged for fresh medium supplemented with 10% CCK-8 (HYCEZMBIO, China). Absorbance readings (450 nm) after 1 h (37°C) were used to generate growth curves.

### 2.9 Immunoprecipitation

First, wash A/G protein magnetic beads (MCE) with NP40 lysis buffer (Solarbio, China) and divide them into two tubes. Add 5 µg of the specific antibody to one tube, which is marked as the IP tube, and add IgG antibody (MCE) to the other tube, marked as the IgG tube. Rotate the tubes at 4°C overnight. On the next day, wash the cells with precooled PBS. Then, lyse the cells in IP cell lysis buffer (Epizyme Biotech, China) containing protease inhibitors (PMSF), and incubate on ice for 30 minutes. Centrifuge at 14,000 g for 15 minutes, and collect the supernatant as the protein lysate. Use a magnetic stand to separate the beads from the supernatant and wash the beads with NP40 buffer, discarding the supernatant. Then, divide the protein lysate equally and add it to both the IP and IgG tubes. Rotate the tubes at 4°C overnight. On the third day, use a magnetic stand to separate the beads from the supernatant, discard the supernatant, and gently wash the beads with NP40 buffer. Add 40 µL of NP40 lysis buffer and 10 µL of 5x SDS sample buffer (Servicebio, China) to each tube, and heat at 95°C for 5 minutes. Use a magnetic stand to separate the beads from the supernatant, and collect the supernatant for Western blot analysis.

### 2.10 Statistical analysis

Statistical analyses were performed using R 4.2.2 and GraphPad Prism 8.0. Paired t-tests, one-way ANOVA, or two-way ANOVA were used to assess differences between groups. Continuous variables were expressed as mean ± SD. All experiments were repeated at least three times. Statistical significance was set at P < 0.05.

## 3 Results

### 3.1 Differential gene expression analysis and functional enrichment

**Figure 1:**
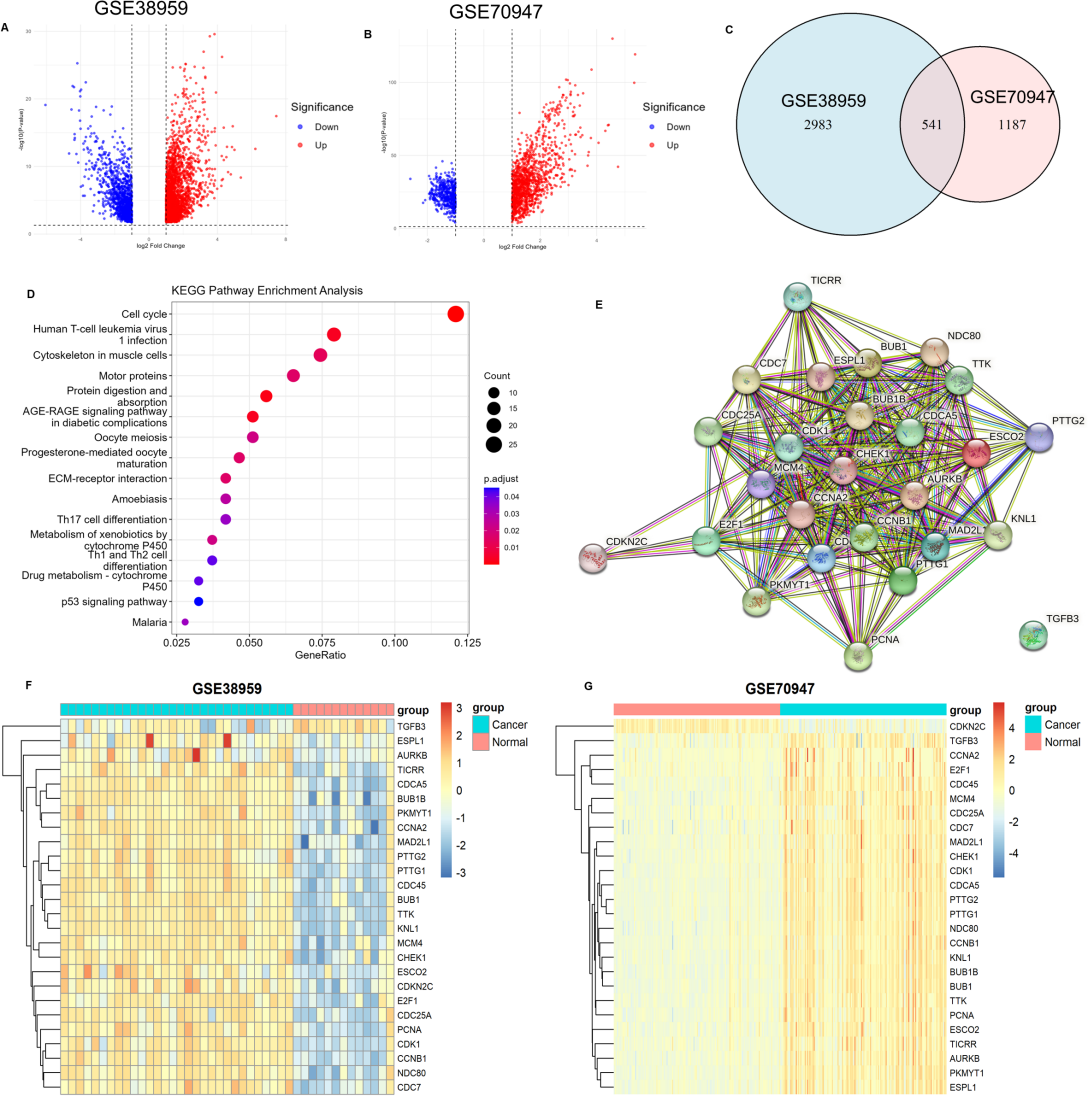
Differential Gene Expression Analysis and Functional Enrichment. **(A)** Volcano plot of differentially expressed genes in the GSE38959 dataset (n=43, 13 adjacent normal tissue samples, 30 tumor tissue samples; DESeq2). **(B)** Volcano plot of differentially expressed genes in the GSE70947 dataset (n=296, 148 adjacent normal tissue samples, 148 tumor tissue samples; DESeq2). **(C)** Venn diagram of differentially expressed genes in the GSE38959 and GSE70947 datasets. **(D)** KEGG functional enrichment analysis of common differentially expressed genes. **(E)** Protein-protein interaction network of genes enriched in the cell cycle. **(F)** Heatmap showing the distribution of genes enriched in the cell cycle in the GSE38959 dataset. **(G)** Heatmap showing the distribution of genes enriched in the cell cycle in the GSE70947 dataset.

We first performed differential gene expression analysis on the sequencing data of breast cancer samples and normal breast samples from the GSE38959 and GSE70947 datasets, respectively. In the GSE38959 dataset, 2317 genes were upregulated and 1207 were downregulated (**Figure 1A**), while in the GSE70947 dataset, 1239 genes were upregulated and 489 were downregulated (**Figure 1B**). There were 541 common differentially expressed genes shared between the two datasets (**Figure 1C**). KEGG functional enrichment analysis of these common differentially expressed genes showed that they were enriched in pathways such as the cell cycle, Human T-cell leukemia virus 1 infection, and cytoskeleton in muscle cells (**Figure 1D**).

Among these, the cell cycle pathway contained the highest number of enriched genes, totaling 26, including ESCO2. Analysis of the expression distribution of these 26 genes in the GSE38959 and GSE70947 datasets revealed that most were highly expressed in breast cancer tissues and lowly expressed in normal breast tissues (**Figures 1F, G**). A protein-protein interaction network constructed using the STRING database demonstrated that ESCO2 interacts with multiple cell cycle regulators (e.g., CDK1, CHEK1, MCM4, CCNB1) (**Figure 1E**). Of particular interest are CDK1 (the central cell cycle kinase) and CHEK1 (a critical DNA damage checkpoint regulator). These findings suggest that ESCO2 may function as a potential key regulator in the cell cycle.

### 3.2 GSEA and validation of ESCO2 expression differences

**Figure 2:**
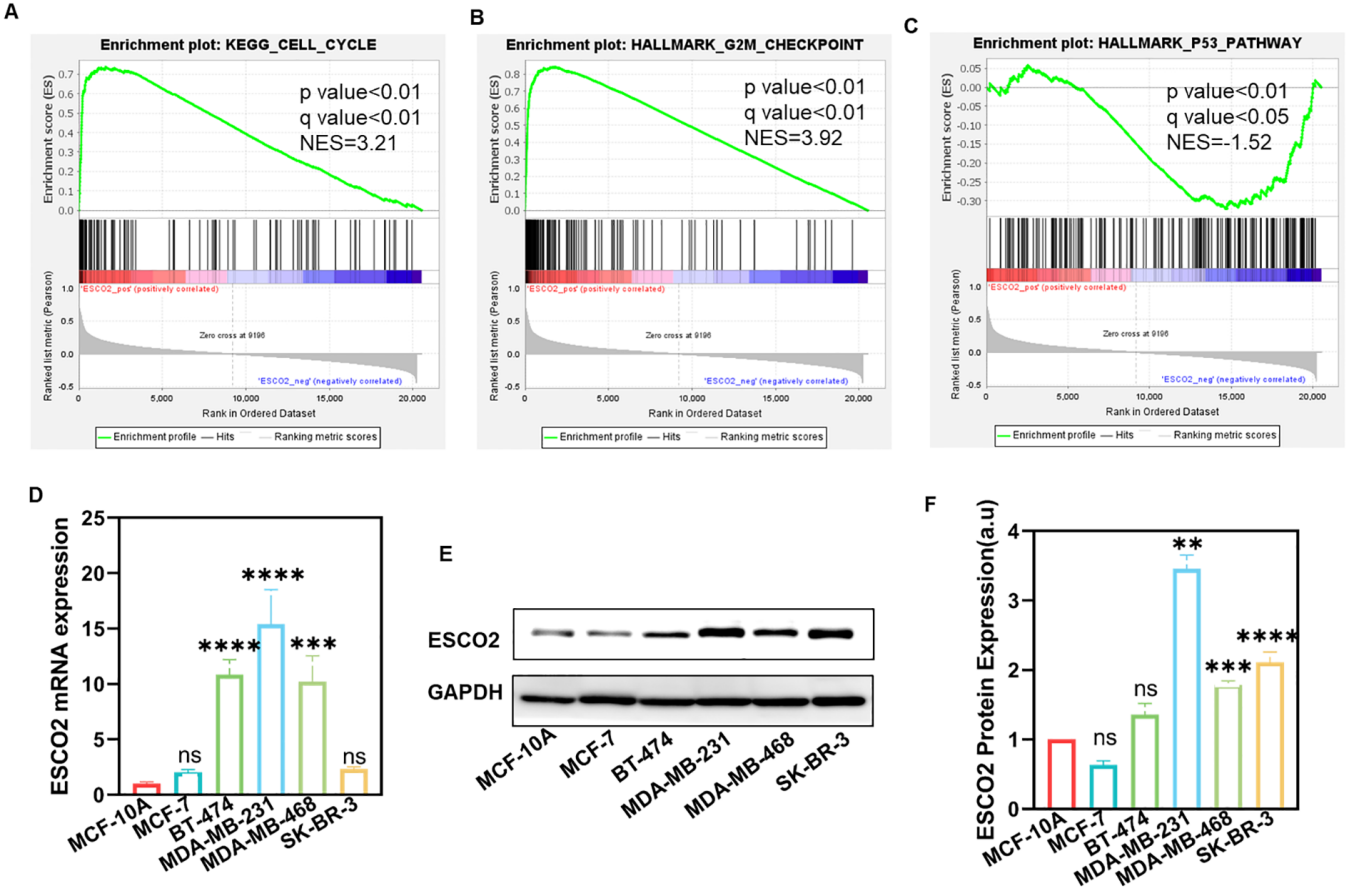
GSEA and the expression of ESCO2 in breast cancer cell lines. **(A-C)** Single-gene GSEA results of ESCO2. **(D)** ESCO2 mRNA expression in normal breast cells (MCF-10A) and various breast cancer cell lines (n=3, One-way ANOVA). **(E, F)** Protein expression levels and relative quantification of ESCO2 in MCF-10A cells and breast cancer cell lines (n=3, One-way ANOVA). (ns: *p*>0.05, **p < 0.01, ***p < 0.001, ****p < 0.0001).

Gene Set Enrichment Analysis (GSEA) further indicated that higher ESCO2 expression was enriched in the cell cycle and G2/M checkpoint (**Figures 2A, B**), while lower ESCO2 expression was enriched in the P53 pathway (**Figure 2C**). These findings suggest that ESCO2 may contribute to the development and progression of breast cancer by regulating the cell cycle and the P53 signaling pathway.

We further validated the differential expression of ESCO2 in cell lines. RT-qPCR results revealed that ESCO2 mRNA was expressed at low levels in the normal breast cell line MCF10A but was significantly upregulated in breast cancer cell lines BT-474, MDA-MB-231, and MDA-MB-468 (**Figure 2D**). In contrast, no significant difference in ESCO2 mRNA expression was observed in MCF-7 and SK-BR-3 breast cancer cells compared to normal breast cells (**Figure 2D**). At the protein level, ESCO2 was upregulated in MDA-MB-231, MDA-MB-468, and SK-BR-3 cells compared to MCF-10A, while no significant difference was detected in MCF-7 and BT-474 cells (**Figures 2E, F**). These findings further confirm the elevated expression of ESCO2 in breast cancer and provide a foundation for subsequent experiments.

### 3.3 Overexpression of ESCO2 promotes DNA replication

**Figure 3:**
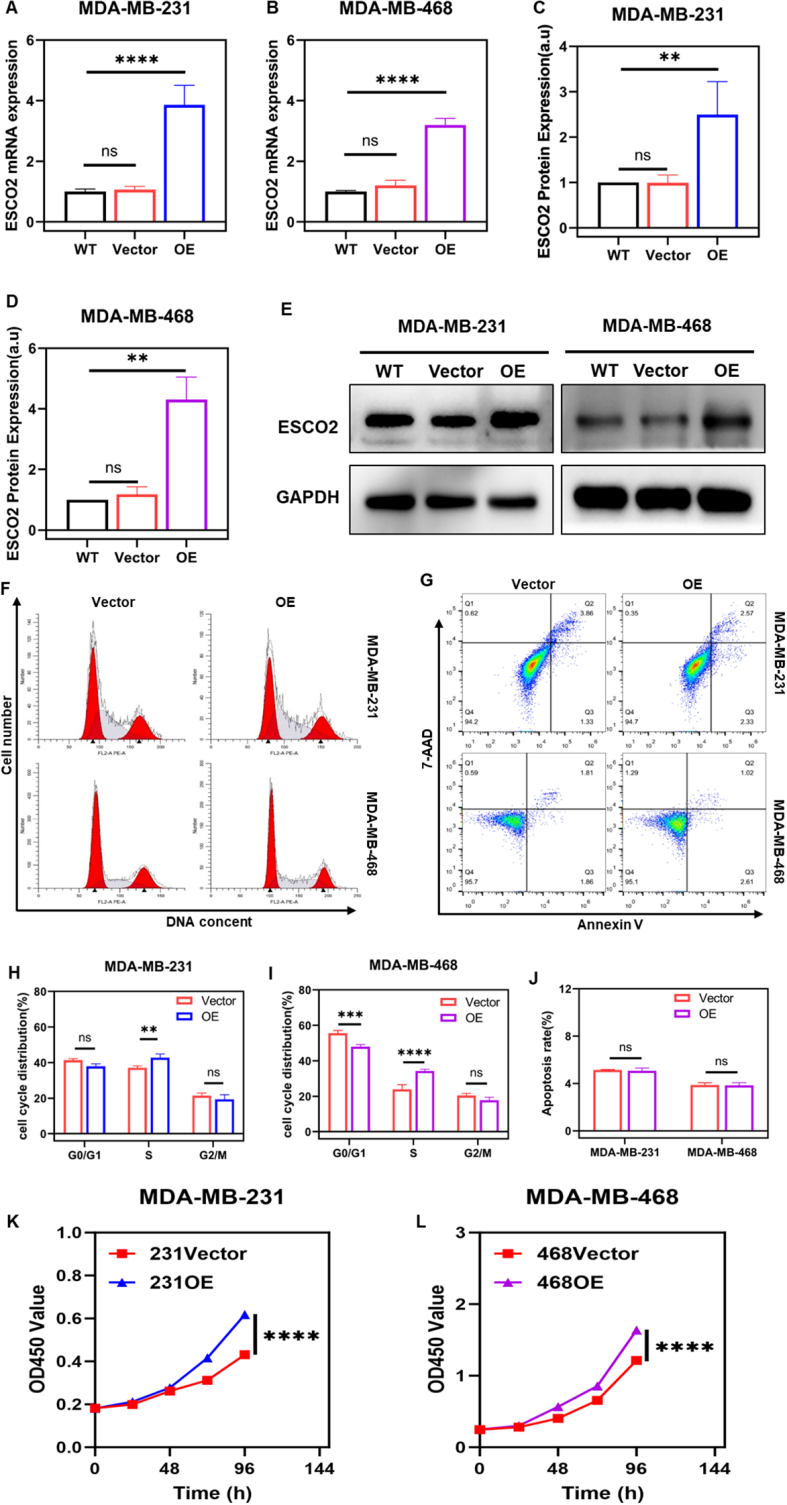
Overexpression of ESCO2 promotes DNA replication. **(A, B)** Validation of lentiviral transfection efficiency for ESCO2 overexpression via qRT-PCR in MDA-MB-231 and MDA-MB-468 cells (n=3, One-way ANOVA). **(C-E)** Validation of lentiviral transfection efficiency for ESCO2 overexpression via Western blot in MDA-MB-231 and MDA-MB-468 cells (n=3, One-way ANOVA). **(F, H, I)** Flow cytometry analysis of cell cycle distribution in MDA-MB-231 and MDA-MB-468 cells overexpressing ESCO2 (n=3, unpaired t test). **(G, J)** Flow cytometry analysis showing the effects of ESCO2 overexpression on apoptosis in MDA-MB-231 and MDA-MB-468 cells (n=3, unpaired t test). **(K, L)** CCK8 assay showed that ESCO2 overexpression promoted cell proliferation (n=3, Two-way ANOVA). (ns: *p* > 0.05, **p < 0.01, ***p < 0.001, ****p < 0.0001).

We selected the MDA-MB-231 and MDA-MB-468 breast cancer cell lines for *in vitro* experiments due to the relatively consistent mRNA and protein expression levels of ESCO2 in these cells. The cells were transfected with control lentivirus or lentivirus overexpressing ESCO2. Transfection efficiency was validated using RT-qPCR and western blot analysis. RT-qPCR results showed that, compared to the wild-type (WT) group, the mRNA expression level of ESCO2 in the vector control group exhibited no significant change (**Figures 3A, B**). In contrast, the overexpression (OE) group demonstrated a significantly higher level of ESCO2 mRNA compared to both the WT and vector control groups (**Figures 3A, B**). Similarly, western blot results revealed that the protein level of ESCO2 in the vector control group showed no significant change compared to the WT group (**Figures 3C-E**). However, the OE group exhibited a markedly increased protein expression of ESCO2 (**Figures 3C-E**).

Subsequently, we employed flow cytometry to assess the impact of ESCO2 overexpression on the cell cycle and apoptosis in breast cancer cells. The results demonstrated that in MDA-MB-231 cells, compared to the vector control group, overexpression of ESCO2 led to a significant increase in the proportion of cells in the S phase of DNA replication, while the proportions of cells in the G0/G1 and G2/M phases slightly decreased, though not significantly (**Figures 3F, H**). Similarly, in MDA-MB-468 cells, overexpression of ESCO2 resulted in a marked increase in the number of cells in the S phase, accompanied by a relative reduction in G0/G1 phase cells, with no significant change observed in the G2/M phase (**Figures 3F, I**).

Flow cytometry analysis of apoptosis revealed that in MDA-MB-231 cells, the average total apoptosis rate was 5.14% in the vector control group and 5.07% in the overexpression group, indicating a slight decrease in apoptosis rate, but the difference was not statistically significant (**Figures 3G, J**). In MDA-MB-468 cells, the average total apoptosis rate was 3.87% in the control group and 3.84% in the overexpression group, with no statistically significant difference observed (**Figures 3G, J**).

To further validate the effect of ESCO2 on DNA synthesis in breast cancer cells, we examined cell proliferation changes after ESCO2 overexpression using the CCK-8 assay. The CCK-8 results demonstrated that compared to the vector control group, ESCO2 overexpression accelerated the proliferation rate in both breast cancer cell lines (**Figures 3K, L**).

### 3.4 Knockdown of ESCO2 resulted in cell cycle arrest and promotes apoptosis

**Figure 4:**
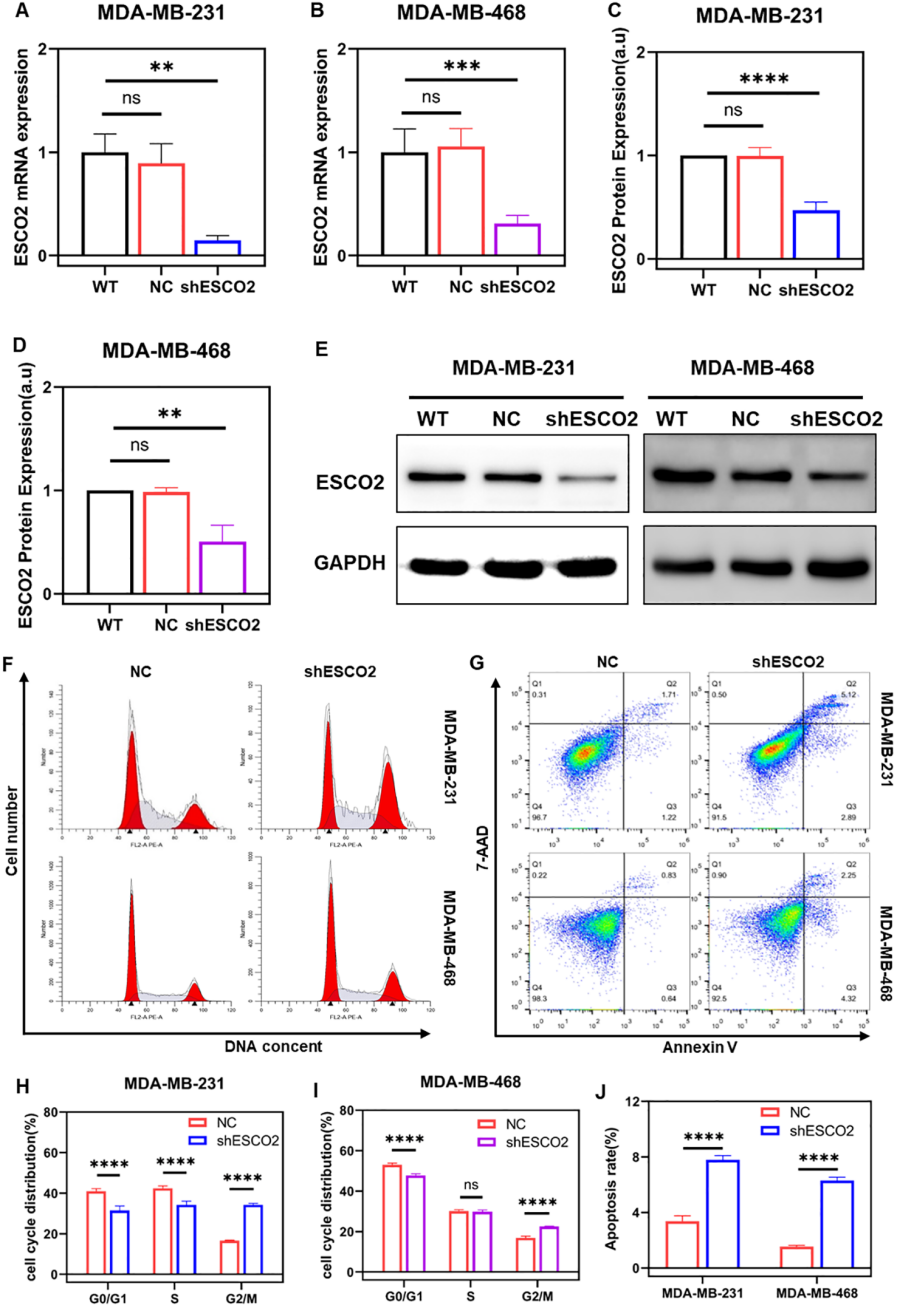
Knockdown of ESCO2 induces cell cycle arrest and promotes apoptosis. **(A, B)** Validation of lentiviral transfection efficiency for ESCO2 knockdown via qRT-PCR in MDA-MB-231 and MDA-MB-468 cells (n=3, One-way ANOVA). **(C-E)** Validation of lentiviral transfection efficiency for ESCO2 knockdown via Western blot in MDA-MB-231 and MDA-MB-468 cells (n=3, One-way ANOVA). **(F, H, I)** Flow cytometry analysis of cell cycle distribution in MDA-MB-231 and MDA-MB-468 cells with ESCO2 knockdown (n=3, unpaired t test). **(G, J)** Flow cytometry analysis showing the effects of ESCO2 knockdown on apoptosis in MDA-MB-231 and MDA-MB-468 cells (n=3, unpaired t test). (ns: *p* > 0.05, **p < 0.01, ***p < 0.001, ****p < 0.0001).

We transfected MDA-MB-231 and MDA-MB-468 breast cancer cells with lentiviruses carrying shESCO2 fragments or negative control (NC). RT-qPCR results showed that the mRNA expression of ESCO2 in the NC group exhibited no significant change compared to the WT group (**Figures 4A, B**). In contrast, the knockdown group (shESCO2) demonstrated significantly lower ESCO2 mRNA expression than both the WT and NC groups (**Figures 4A, B**). Western blot analysis revealed that the protein expression of ESCO2 in the NC group showed no significant change compared to the WT group (**Figures 4C-E**). However, the shESCO2 group exhibited significantly lower ESCO2 protein expression than both the WT and NC groups (**Figures 4C-E**).

Similarly, we used flow cytometry to assess the effects of ESCO2 knockdown on the cell cycle and apoptosis in breast cancer cells. Cell cycle analysis revealed that in MDA-MB-231 cells, ESCO2 knockdown significantly reduced the proportion of cells in the G0/G1 and S phases, while markedly increasing the proportion of cells in the G2/M phase (**Figures 4F, H**). In MDA-MB-468 cells, ESCO2 knockdown led to a significant decrease in G0/G1 phase cells, no significant change in S phase cells, and a notable increase in G2/M phase cells (**Figures 4F, I**).

Apoptosis analysis showed that in MDA-MB-231 cells, the average total apoptosis rate was 3.37% in the NC group and 7.79% in the shESCO2 group, indicating a significant increase in apoptosis (**Figures 4G, J**). Similarly, in MDA-MB-468 cells, the average total apoptosis rate was 1.55% in the NC group and 6.31% in the shESCO2 group, also demonstrating a significant increase in apoptosis (**Figures 4G, J**).

### 3.5 ESCO2 deletion triggers p53-CDK1-dependent cycle arrest and BAX/Bcl2-caspase-mediated apoptosis

**Figure 5:**
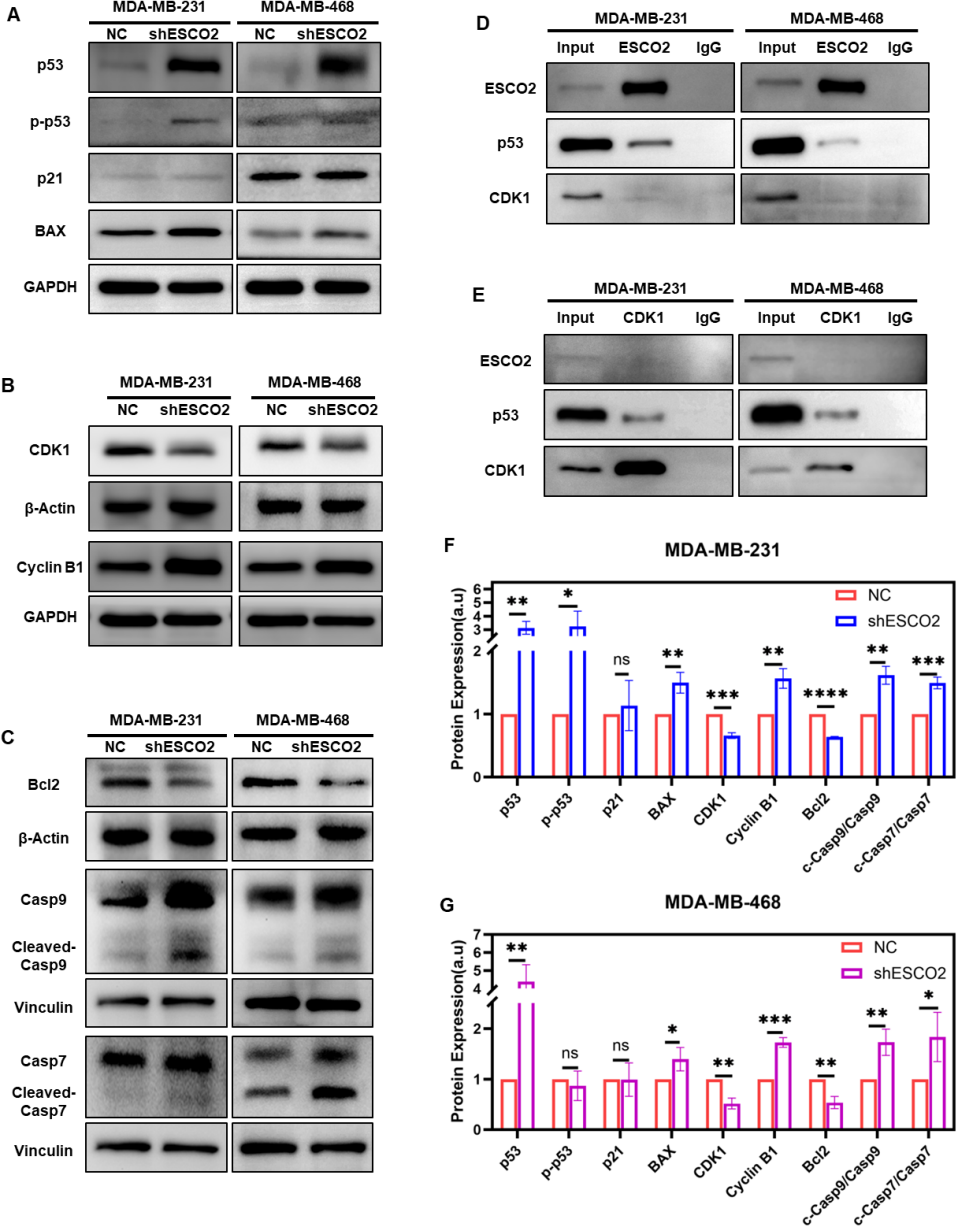
Molecular mechanisms of ESCO2 in regulating cell cycle and apoptosis. **(A)** Western blots showing alterations in p53, p-p53, p21 and BAX expression with ESCO2 knockdown. **(B)** Western blots showing alterations in CDK1 and cyclin B1 expression with ESCO2 knockdown. **(C)** Western blot analysis of Bcl-2, caspase-9, and caspase-7 expression and activation following ESCO2 knockdown. **(D)** Co-IP assays were performed using anti-ESCO2 antibody or IgG control. **(E)** Co-IP assays were performed using anti-CDK1 antibody or IgG control. **(F, G)** Quantitative analysis of protein expression (n=3, unpaired t test). (ns: *p* > 0.05, *p < 0.05, **p < 0.01, ***p < 0.001, ****p < 0.0001).

To further investigate the molecular mechanisms by which ESCO2 affects the cell cycle and apoptosis in breast cancer cells, we assessed the expression changes of related proteins. Based on GSEA analysis suggesting ESCO2’s potential involvement in the p53 pathway, we first analyzed the expression levels of p53 and its downstream effectors. The results showed that, upon ESCO2 knockdown, p53 protein expression was significantly increased in both types of breast cancer cells (**Figures 5A, F, G**). Notably, phospho-p53 (p-p53) levels were markedly increased in MDA-MB-231 cells but remained unchanged in MDA-MB-468 cells (**Figures 5A, F, G**). Further analysis revealed that while p21 expression showed no significant alteration in either cell line, the pro-apoptotic protein BAX was significantly upregulated (**Figures 5A, F, G**).

Given that the CDK1/cyclin B1 complex serves as a critical molecular switch regulating G2/M phase transition, and considering the cell cycle arrest observed in previous flow cytometry experiments, we examined the protein levels of these key cell cycle regulators. Western blot analysis indicated that ESCO2 knockdown significantly reduced CDK1 expression while markedly increasing cyclin B1 levels (**Figures 5B, F, G**).

Additionally, we examined changes in apoptosis-related proteins Bcl-2, caspase-9, and caspase-7. The results showed that, following ESCO2 knockdown, Bcl-2 expression was significantly decreased (**Figures 5C, F, G**), and the ratios of cleaved-caspase-9/caspase-9 and cleaved-caspase-7/caspase-7 were both significantly elevated (**Figures 5C, F, G**).

Considering that both MDA-MB-231 and MDA-MB-468 cells harbor p53 mutations (as documented in the COSMIC database: p.R280K, c.839G>A in MDA-MB-231 and p.R273H, c.818G>A in MDA-MB-468), and that the canonical p53 downstream effector p21 was not activated, we employed co-immunoprecipitation assays to investigate the non-canonical mechanisms underlying ESCO2-mediated cell cycle regulation. The results revealed a direct interaction between ESCO2 and p53 (**Figure 5D**), but no significant binding between ESCO2 and CDK1 (**Figures 5D, E**). Intriguingly, we identified a distinct interaction between CDK1 and p53 (**Figure 5E**), providing novel experimental evidence for the ESCO2-p53-CDK1 regulatory network.

### 3.6 Using siP53 rescued the effect of knockdown ESCO2 on breast cancer

**Figure 6:**
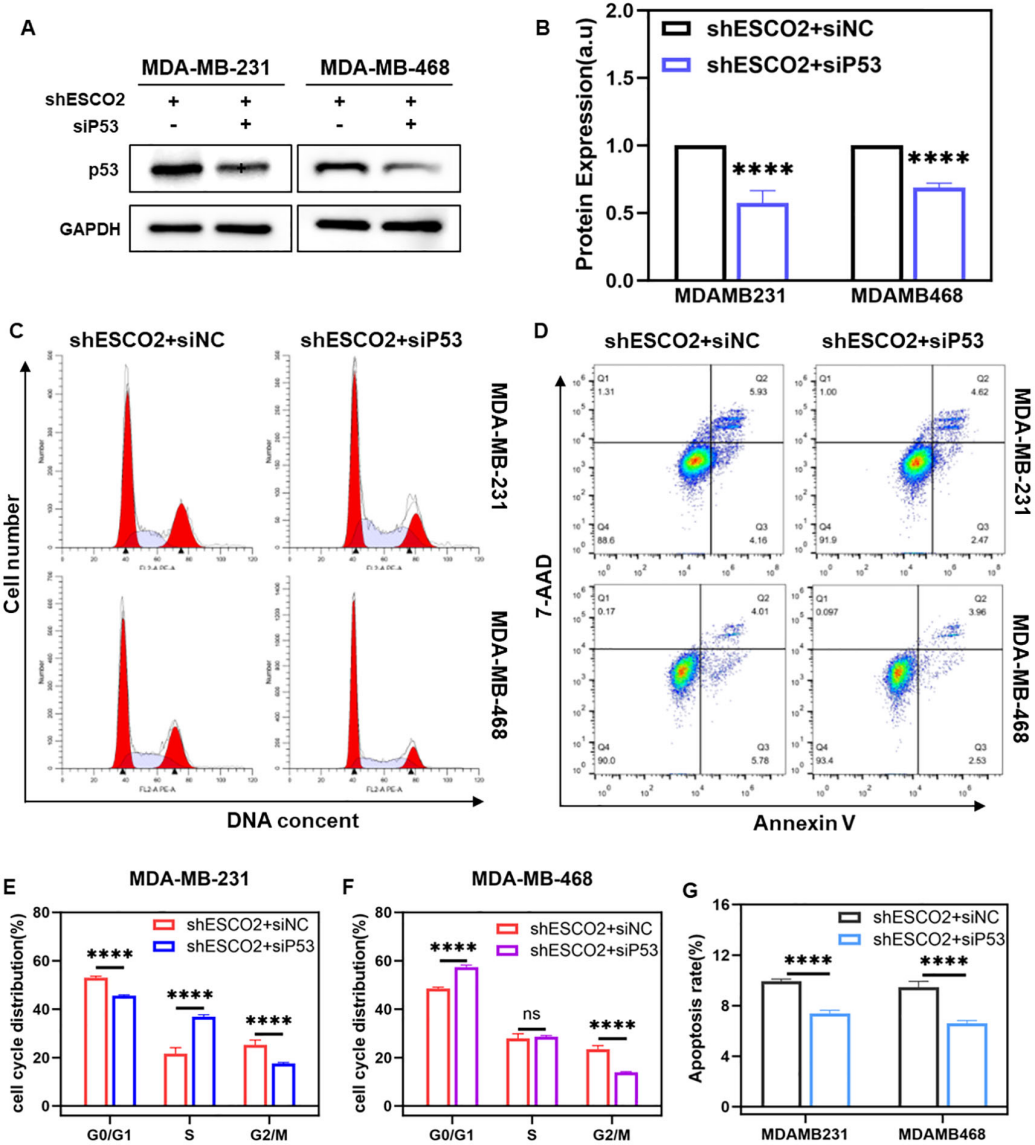
Using siP53 rescued the effect of knockdown ESCO2 on breast cancer. **(A, B)** Western blot shows siRNA inhibits P53 expression (n=3, unpaired t test). **(C, D, F)** The cell cycle distribution of shESCO2 MDA-MB-231 and shESCO2 MDA-MB-468 cells in the siNC and siP53 groups (n=3, unpaired t test). **(E,G)** Apoptosis rate of shESCO2 MDA-MB-231 and shESCO2 MDA-MB-468 cells in siNC and siP53 group (n=3, unpaired t test). (ns: *p* > 0.05, ****p < 0.0001).

To investigate the critical role of p53 in ESCO2-mediated regulation of the cell cycle and apoptosis, we specifically inhibited P53 expression using siRNA. Breast cancer cells were transfected with siP53 or its control siNC. Western blot analysis showed significantly reduced P53 protein expression in the siP53 group compared to the siNC group (**Figures 6A, B**).

Subsequently, we used flow cytometry to assess the effects of P53 inhibition on the cell cycle and apoptosis in stable shESCO2-transfected breast cancer cells. Cell cycle analysis revealed that in MDA-MB-231-shESCO2 cells, compared to the siNC group, the siP53 group exhibited a significant decrease in the proportion of G0/G1 phase cells, an increase in S phase cells, and a notable reduction in G2/M phase cells (**Figures 6C, F**). In MDA-MB-468-shESCO2 cells, the siP53 group showed an markedly increase in G0/G1 phase cells, no significant change in S phase cells, and a significant decrease in G2/M phase cells compared to the siNC group (**Figures 6D, F**).

Apoptosis analysis demonstrated that in MDA-MB-231-shESCO2 cells, the average total apoptosis rate was 9.93% in the siNC group and 7.38% in the siP53 group, indicating a statistically significant reduction in apoptosis (**Figures 6E, G**). Similar results were observed in MDA-MB-468-shESCO2 cells, where the average total apoptosis rate was 9.47% in the siNC group and 6.59% in the siP53 group, showing a statistically significant decrease in apoptosis (**Figures 6E, G**). Therefore, siP53 successfully reversed the cell cycle arrest and apoptosis induced by ESCO2 knockdown in breast cancer cells.

## 4 Discussion

The incidence rate of BRCA remains notably high. Especially triple-negative breast cancer, which poses a particularly severe threat due to its high malignancy, aggressive invasion and elevated recurrence rate (12). This underscores the urgent need for more effective treatment strategies, with the identification of novel therapeutic targets representing a promising approach. Among these, the application of cell cycle inhibitors, such as CDK4/6 inhibitors, in breast cancer treatment has provided valuable insights (13, 14). By analyzing differentially expressed genes between breast cancer samples and normal breast samples across multiple GEO datasets, this study identified that genes associated with the cell cycle pathway constituted the largest proportion. Further protein-protein interaction (PPI) network analysis suggested that ESCO2 may serve as a potentially important molecule in this pathway, although it is not a hub protein. However, STRING database predictions revealed its interactions with multiple core cell cycle regulators (e.g., CDK1, CHEK1, MCM4, CCNB1, etc.), indicating that its potential functional relevance warrants in-depth investigation. Among these, CDK1 and CHEK1 are particularly noteworthy. CDK1 is a core kinase regulating G2/M phase transition and is closely associated with mitotic progression (15). CHEK1 functions as a DNA damage response factor involved in maintaining genomic stability (16).

Numerous studies have reported elevated ESCO2 expression in various tumor types, often correlating with poor clinical outcomes (17, 18). However, the biological functions of ESCO2 in breast cancer remain poorly understood. To our knowledge, few studies have systematically investigated its biological role specifically in breast cancer, which underscores the novelty of our focus on ESCO2 in this context. This study first predicted the potential pathways through which ESCO2 might function using GSEA, and then examined the differential expression of ESCO2 in various breast cancer cell lines compared to normal breast cells. Our study revealed significant variations in ESCO2 expression at both mRNA and protein levels among different breast cancer cell lines. This heterogeneity may stem from their distinct molecular subtypes (MCF-7 as ER+/HER2-; BT474 and SKBR3 as HER2+; MDA-MB-231 and MDA-MB-468 as triple-negative), which exhibit intrinsic differences in gene expression profiles. Furthermore, cell line-specific genetic mutations, copy number variations, or epigenetic modifications may collectively contribute to the differential ESCO2 expression patterns. The underlying regulatory mechanisms warrant further investigation.

To assess the functional consequences of ESCO2 in breast cancer cells, we used lentiviral transduction to either overexpress or knock down its expression, and subsequently examined its effects on cell cycle progression and apoptosis. Our flow cytometry analysis revealed that overexpression of ESCO2 increases the S phase in breast cancer cells, which is consistent with previous studies. Research has shown that ESCO2 modifies adhesion proteins during the S phase, thereby stabilizing sister chromatid cohesion and facilitating gene transcription (19). Moreover, CCK-8 assays demonstrated that overexpression of ESCO2 significantly enhanced the proliferation rate of breast cancer cells. Therefore, overexpression of ESCO2 promotes DNA replication in breast cancer cells. In contrast, our findings indicate that knockdown of ESCO2 leads to an extended G2/M phase in the cell cycle of breast cancer cells. We hypothesize that ESCO2 knockdown disrupts sister chromatid cohesion, causing cells to prematurely enter the G2 phase in a defective state, which impedes progression to the M phase and ultimately results in cell cycle arrest.

The tumor suppressor p53 plays pivotal roles in cell cycle regulation, DNA repair, and apoptosis (20). However, p53 is frequently mutated in cancer cells, leading to functional loss or alteration. Intriguingly, some cellular functions may still be mediated through mutant p53 or alternative pathways (21, 22). The cell lines used in this study harbor p53 mutations. We observed that while ESCO2 knockdown significantly increased both total and phosphorylated p53 (p-p53) levels in MDA-MB-231 cells, no such increase in p-p53 was detected in MDA-MB-468 cells, indicating differential p53 activation status. Notably, the canonical downstream effector p21 remained unactivated in both cell lines, suggesting that ESCO2-mediated cell cycle arrest occurs independently of p21.

The CDK1/cyclin B1 complex serves as the master regulator of G2/M checkpoint control (23). Our data demonstrate that ESCO2 knockdown suppresses CDK1 while paradoxically increasing cyclin B1 accumulation. Co-immunoprecipitation experiments revealed that ESCO2 may orchestrate G2/M arrest through formation of an ESCO2-p53-CDK1 regulatory axis. These findings suggest that although mutant p53 loses canonical cell cycle regulatory functions, it may still inhibit CDK1 activity through direct binding, thereby inducing G2/M arrest.

Our results demonstrated that while ESCO2 overexpression showed no significant effect on apoptosis, its knockdown markedly increased apoptotic rates in breast cancer cells - consistent with previous observations in gastric and hypopharyngeal carcinomas (11, 24). This suggests a context-dependent regulation of apoptosis by ESCO2 (e.g., only evident under knockdown-induced cellular stress).

In both cell lines, ESCO2 depletion activated BAX. Given their differential p53 activation status, we propose this activation likely occurs indirectly through the p53-CDK1 axis rather than via direct p53-mediated regulation. Importantly, we observed concomitant downregulation of the anti-apoptotic protein Bcl-2 and upregulation of both caspase-9/caspase-7 and their cleaved forms (cleaved-caspase-9/7). The increased BAX/BCL-2 ratio represents a hallmark event in mitochondrial apoptosis (25, 26), and caspase-9, the initiator of mitochondrial apoptosis Furthermore, activation of caspase-9 - the initiator of mitochondrial apoptosis (27) - and downstream caspase family proteins strongly indicates engagement of the intrinsic apoptotic pathway. Thus, ESCO2 knockdown likely triggers apoptosis through the p53-CDK1 axis-mediated cell cycle arrest, which further activates the BAX/Bcl-2-caspase signaling cascade.

To further elucidate p53’s role, we employed siRNA-mediated p53 suppression. P53 inhibition effectively rescued ESCO2 knockdown-induced G2/M arrest and significantly reduced apoptosis rates, confirming the essential role of the ESCO2-p53-CDK1 axis in mediating these effects.

In summary, our study demonstrates that ESCO2 is highly expressed in breast cancer and serves as a potential master regulator of cell cycle progression in breast cancer cells. Targeting ESCO2 induces G2/M arrest via the p53-CDK1 axis while activating the pro-apoptotic BAX/Bcl-2-caspase cascade. These findings provide novel mechanistic insights into breast cancer pathogenesis and suggest ESCO2 as a potential therapeutic candidate. Further *in vivo* and translational studies, including investigations in normal mammary epithelial cells, are warranted to evaluate target specificity and clinical potential.

## Data Availability

The original contributions presented in the study are included in the article/supplementary material. Further inquiries can be directed to the corresponding author.
